# Effects of multi-frequency ultrasound on the freezing rates, quality properties and structural characteristics of cultured large yellow croaker (Larimichthys crocea)

**DOI:** 10.1016/j.ultsonch.2021.105657

**Published:** 2021-07-01

**Authors:** Xuan Ma, Jun Mei, Jing Xie

**Affiliations:** a College of Food Science and Technology, Shanghai Ocean University, Shanghai 201306, China; b National Experimental Teaching Demonstration Center for Food Science and Engineering Shanghai Ocean University, Shanghai 201306, China; c Shanghai Engineering Research Center of Aquatic Product Processing and Preservation, Shanghai 201306, China; d Shanghai Professional Technology Service Platform on Cold Chain Equipment Performance and Energy Saving Evaluation, Shanghai 201306, China

**Keywords:** Large yellow croaker, Assisted freezing, Multi-frequency ultrasound, Freezing rate, Quality properties

## Abstract

This research evaluated the effects of multi-frequency ultrasound assisted freezing (UAF) on the freezing rate, structural characteristics, and quality properties of cultured large yellow croaker. The freezing effects with triple ultrasound-assisted freezing (TUF) at 20, 28 and 40 kHz under 175 W was more obvious than that of single ultrasound-assisted freezing (SUF) at 20 kHz and dual ultrasound-assisted freezing (DUF) at 20 and 28 kHz. The results showed that UAF significantly increased the freezing rate and better preserved the quality of frozen large yellow croaker samples. Specifically, the quality parameters of the TUF-treated samples were closer to those of the fresh samples, with greater texture characteristics, a larger water holding capacity (lower thawing loss and cooking loss), lower K values and lower thiobarbituric acid reactive substances values. Light microscopy observation images revealed that the ice crystals formed by TUF were fine and evenly distributed, resulting in less damage to the frozen large yellow croaker samples. Therefore, multi-frequency UAF could improve the quality properties of the large yellow croaker samples.

## Highlights

• Multi-frequency ultrasound assisted freezing accelerated the freezing rate.
• Increase in the number of ultrasonic frequencies enhanced the cavitation effects.
• TUF treated samples retained better quality attributes.
• Light microscopy observation was adopted to study the microstructures of frozen fish.

## 1 Introduction

Large yellow croaker (Larimichthys crocea) is a marine fish with delicious meat and high nutritional value that is considered to be one of the most commercially and economically valuable fish in China [1]. Freezing could reduce the spoilage and extend the shelf life of large yellow croaker samples by inhibiting the enzymatic and water activities [2]. The size of the ice crystals size formed during the freezing process is an important parameter that affects the microstructure and thawing parameters of frozen fish. Traditional freezing methods, such as air blast freezing [3], direct-contact freezing [4], immersion freezing [5], normally have a low freezing rate, which results in the generation of large and unevenly distributed ice crystals and causes destruction to the muscle tissue [6], [7]. Therefore, slow freezing is averse to maintaining the integrity of muscle tissue. Therefore, it is necessary to develop an efficient and cost-effective freezing method to accelerate the freezing rates and regulate the ice nucleation processes to better maintain the quality of frozen large yellow croaker samples.

In the past few years, ultrasound-assisted immersion freezing (UIF) has become one of the research hotspots because it can accelerate freezing rates and shorten freezing times [8], [9]. Ultrasonic treatment could produce cavitation and microstreaming to shorten the freezing time during the UIF process. The cavitation bubbles could be used as the primary ice nuclei and the mechanical force generated by the bursting of the cavitation bubbles breaks the ice crystals into smaller pieces, which again act as primary nuclei to increase the freezing rate [10]. At present, most studies on UIF carried out with mono-frequency ultrasonic treatment. For example, Sun et al. [42] evaluated the effects of UIF on the freezing rate and quality of common carp with different ultrasonic powers (125, 150, 175, 200 and 225 W) and showed that UIF at 175 W had the fastest freezing rate and formed smaller and more uniform ice crystals. Zhang et al. [11] used constant low-frequency ultrasound (30 kHz) with different ultrasonic powers (120, 180, 240 and 300 W) to freeze porcine longissimus muscles and found that UIF at 180 W had the shortest total freezing time and less structural damage. However, higher ultrasonic power (240 and 300 W) resulted in larger ice crystals and weakening of the physical structure of the muscle. High mono-frequency ultrasonic power treatment could damage the muscle structure and cause the cells to rupture [12]. Ma et al. [13] reported that dual-frequency flat ultrasonic treatment had a wider range of uniform energy dissipation than mono-frequency flat ultrasonic treatment. Zhu et al. [14] evaluated the effects of multi-frequency ultrasonic treatment on the freezing rates and quality attributes of potatoes and found that multi-frequency ultrasonic treatment could give better results than single-frequency ultrasonic treatment on the freezing rate, microstructure and quality properties.

Based on the abovementioned research results, we hypothesized that multi-frequency UIF could reduce fish damage during the freezing process. However, little is known about the effect of UIF at different frequencies on the quality properties of large yellow croaker. Therefore, the present research evaluated the effects of UIF at different frequencies on accelerating the freezing rate of large yellow croaker samples during the freezing process and their effects on the quality properties including thawing loss, cooking loss, pH value, water holding capacity (WHC), texture profile analysis (TPA), colour, water distribution and migration, thiobarbituric acid reactive substances (TBARS), ATP-related compounds, and structural characteristics.

## 2 Materials and methods

### 2.1 Experimental procedure

**Fig. 1:**
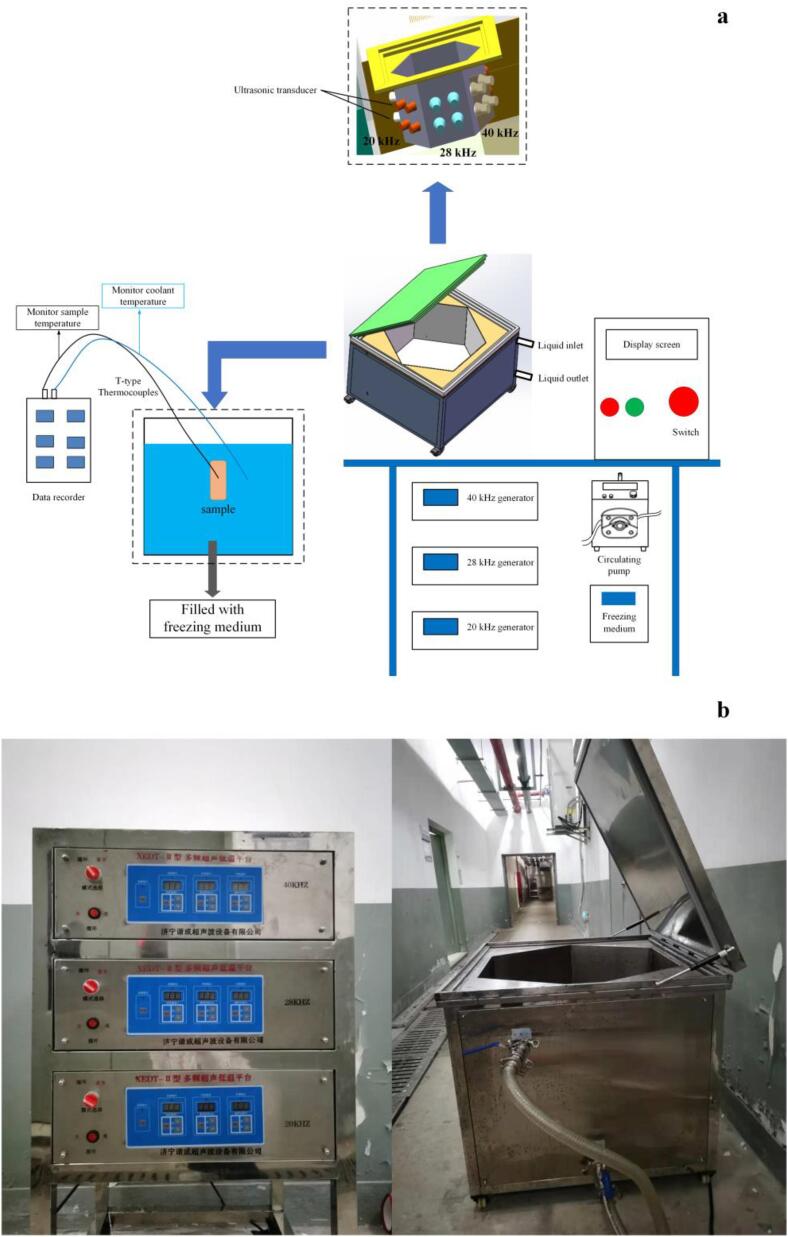
Schematic diagrams of the multi-frequency ultrasound assisted freezing system.

**Fig. 2:**
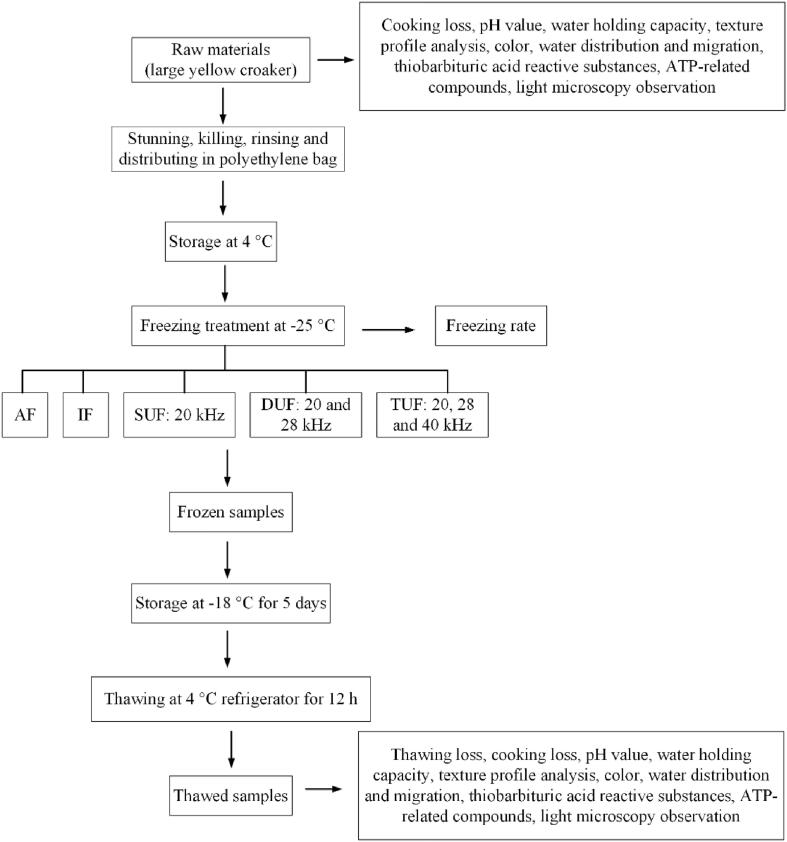
Flow diagram of experimental step. AF, air freezing; IF, immersion freezing; SUF, single-ultrasound assisted freezing; DUF, dual-ultrasound assisted freezing; TUF, triple-ultrasound assisted freezing.

Fresh cultured large yellow croaker samples with an average weight of 550 g ± 25 g were rinsed with normal saline and each fish was packaged separately in a polyethylene bag. The present study took 20 kHz as the main line and superimposed frequencies on the basis of single-ultrasound frequency at 20 kHz to study the effect of ultrasound with dual-ultrasound frequencies at 20 and 28 kHz and triple-ultrasound frequencies at 20, 28 and 40 kHz on the freezing process of large yellow croaker, referring to the methods of Zhu et al. [14] and Wang et al. [15]. Therefore, the fish were randomly divided into five batches as follows: air freezing (AF), immersed freezing (IF), immersed freezing associated with single-ultrasound frequency (SUF) at 20 kHz, immersed freezing associated with dual-ultrasound frequencies (DUF) at 20 and 28 kHz and immersed freezing associated with triple-ultrasound frequencies (TUF) at 20, 28 and 40 kHz. The multi-frequency ultrasound assisted freezing instrument (Fig. 1) was designed by our research team and made by Xiecheng Ultrasonic Equipment Co., Ltd. (Jining, Shandong, China). The ultrasonic system consists of a hexahedral ultrasonic processing system (side length: 60 cm) and three ultrasound transducers. A 29.3% calcium chloride brine solution (w/v) was used as the coolant in the UIF system and the coolant temperature was kept at −25.0 ± 0.5 °C using a pump. The UIF treatment was turned on for 30 s and then off for 30 s off to reduce the negative thermal effects. The temperatures of the large yellow croaker samples were recorded in real-time by a network multi-point temperature acquisition instrument (Fluke 2640A, Fluke Electronic Instrument Co., Ltd, USA) connected to a T-type thermocouple. When the central temperature of the large yellow croaker reached −18 °C, all of the samples were transferred to a thermotank and stored at −18 °C for 5 days. Fig. 2 shows the flow chart of the experimental device.

### 2.2 Thawing loss

The thawing loss of large yellow croaker samples was determined according to Sun et al. [16]. The samples were thawed with cooled running water until the central temperature reached to 4 °C. The water on the surface was absorbed with filter paper. The thawing loss was calculated by the following equation:

$$Thawing\;loss\left(\%\right)=\frac{W_{0}-W_{1}}{W_{0}}\times 100\%$$

W_0_: the weight before thawing (W_0_) and
W_1_: the weight after thawing (W_1_).

### 2.3 Cooking loss

The thawed large yellow croaker samples were cut into 3 cm × 3 cm × 1 cm pieces and packaged separately in polyethylene bags. Then they were cooked at 75 °C in a water bath for 15 min and the water on the surface was absorbed with filter paper. The cooking loss was calculated by the following equation:

$$Cooking\;loss\left(\%\right)=\frac{W_{0}-W_{1}}{W_{0}}\times 100\%$$

W_0_: the weight before cooking (W_0_) and
W_1_: the weight after cooking (W_1_).

### 2.4 pH value

In brief, 5.0 g of minced large yellow croaker flesh was homogenized with 45 mL of deionized water. The homogenate was centrifuged at 3, 040 × g for 15 min at 4 °C. The pH value of the supernatant was determined using a pH meter (PB-10, Sartorius, Germany).

### 2.5 WHC determination

The WHC was determined by the centrifugation method [17] and is expressed as the water retention rate of the large yellow croaker after centrifugation (wet weight basis).

### 2.6 TPA

The thawed dorsal muscle of large yellow croaker was cut into 3 cm × 2 cm × 1.5 cm pieces, and the TPA was determined using a TA. XT Plus texture analyser (Stable Micro Systems, Ltd., Godalming, Surrey, UK) with a P/5 probe [18]. The test speed was 1 m/s, and the sample deformation was 50%. A minimum of 6 points were tested for each sample.

### 2.7 Colour

The colour of the thawed large yellow croaker samples was measured using a colorimeter (CR-400, Konica Minolta, Tokyo, Japan) as proposed by Shi et al. [19].

### 2.8 Water distribution and migration

The distribution and migration of water from large yellow croaker samples were evaluated through proton relaxation experiments using a low field nuclear magnetic resonance (LF-NMR) analyser (Niumag MesoMR23-60H.I, Suzhou, China) with a proton resonance frequency of 21 MHz (corresponding to the pulse sequence of Carr-Purcell-Meiboom-Gill) [20]. The dorsal muscle of the samples was cut into 3 cm × 2 cm × 1.5 cm pieces (approximately 5 g) and wrapped with polyethylene film. For each measurement, 16 scans were performed with 3000 echoes.

### 2.9 Determination of TBARS

TBARS was monitored according to the method of Vale et al. [21] and expressed as mg of malonaldehyde (MDA)/kg of large yellow croaker sample. Five grams of flesh and 20 mL of 20% TBA solution were homogeneously mixed and allowed to stand for 1 h. Then, the mixture was centrifuged at 11, 960 × g at 4 °C for 10 min, and the supernatants were collected. Five millilitres of collected supernatant was mixed with 5 mL of thiobarbituric acid (TBA, 0.02 M) and boiled for 40 min. Then the mixture was immediately transferred to an ice bath, and the absorbance was measured at 532 nm.

### 2.10 Evaluation of ATP-related compounds

According to Karim et al. [22], ATP-related compounds, including adenosine triphosphate (ATP), adenosine diphosphate (ADP), adenosine monophosphate (AMP), inosine monophosphate hypoxanthine (IMP), riboside (HxR) and hypoxanthine (Hx), were determined by HPLC (Waters 2695, Milford, USA) coupled with a Shim-pack VP-ODS C18 column. The K value was calculated by the following equation:

$$K\;value\left(\%\right)=\frac{HxR+Hx}{ATP+ADP+AMP+IMP+HxR+Hx}\times 100$$

### 2.11 Light microscopy observation

Portions of 5 mm × 5 mm × 5 mm large yellow croaker dorsal muscle were sampled and placed in Carnoy fixative [ethanol: glacial acetic acid (v/v) = 3:1] for 24 h. The samples were dehydrated stepwise with gradient ethanol series and placed into xylene to perform transparent processing. Then, the samples were embedded in paraffin to obtain 1 cm^3^ blocks and sectioned with a slicing machine (Leica, Germany). The slices were stained by the haematoxylin-eosin method and observed with a light microscope (Eclipse E200 biological microscope, Nikon Instruments Co. Ltd., Tokyo, Japan).

### 2.12 Statistical analysis

Multiple comparisons were performed by one-way analysis of variance (ANOVA) using SPSS 22.0, and the results are expressed as the means ± standard deviation.

## 3 Results and discussion

### 3.1 Freezing rate

**Fig. 3:**
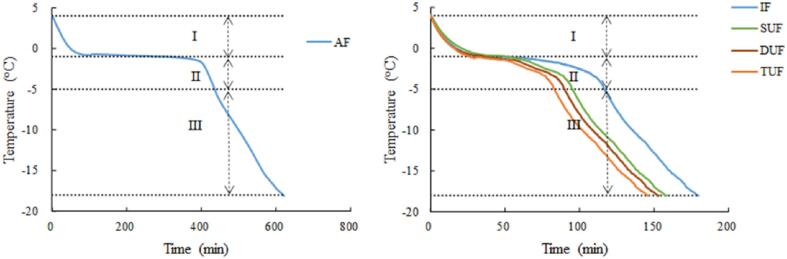
Freezing curves of large yellow croaker samples with different freezing treatments. AF, air freezing; IF, immersion freezing; SUF, single-ultrasound assisted freezing; DUF, dual-ultrasound assisted freezing; TUF, triple-ultrasound assisted freezing. (For interpretation of the references to color in this figure legend, the reader is referred to the web version of this article.)

Rapid freezing can facilitate the formation of small and uniformly distributed ice crystals, which can improve the quality of frozen fish. The time–temperature curves of large yellow croaker samples after different freezing treatments are presented in Fig. 3. The application of ultrasonic treatment during the freezing process of large yellow croaker samples obviously increased the freezing rate. The total freezing time (4 °C to −18 °C) of the AF treated large yellow croaker samples was approximately 300 min, which was more than three times longer than that of the IF treated sample, as the liquid has a higher heat transfer coefficient [11]. TUF treated samples showed a significantly shortened freezing time and an improved freezing rate, which can be attributed to the facilitation of the nucleation process and the improvement of the heat transfer efficiency [14].

The mechanism of ultrasonic treatment for accelerating the freezing process can be summarized into four aspects. (i) A mass of cavitation bubbles form, grow, oscillate, and rupture during the propagation of ultrasound, thereby changing the internal environment of the food substrate, namely, the cavitation effect [23], [24]. Local high pressure (>5 GPa) and extremely high temperature will be generated instantaneously accompanied by the collapse of the cavitation bubbles [25]. In addition, local high pressure can result in an increase in the supercooling degree, providing a great driving force for nucleation [26]. (ii) The microstreaming generated by the ultrasonic cavitation effect can bring about violent collisions of micro-particles in the medium, resulting in strong turbulence [27], [28], which can help to induce nucleation and boost heat and mass transfer efficiency [29]. (iii) Cavitation bubbles can reach the critical size of ice nucleation and act as ice nuclei to promote primary nucleation [9], [30]. (iv) Large dendritic ice crystals can be fragmented into small fragments as a result of the effect of microstreaming, consequently enhancing secondary nucleation [31], [32]. Therefore, ultrasonic treatment during the freezing process could induce the nucleation process and enhance the heat transfer, thus shortening the freezing time. It can also be seen from Fig. 3 that TUF had the fastest freezing rate, followed by DUF, SUF and IF; however, AF had the lowest freezing rate. This could be due to an improvement in cavitation effects, as previously mentioned.

The time–temperature curve could be categorized into three stages: (i) precooling stage (or chilling stage): 4 to −1 °C; (ii) phase transition stage: −1 to −5 °C; and (iii) subcooling stage: −5 to −18 °C [33]. In this research, the freezing process was set to 4 to −1 °C, −1 to −5 °C and −5 to −18 °C. The total freezing time was reduced by 74.33%, 75.38% and 76.49% for SUF, DUF and TUF-treated samples, respectively, compared with the AF treated samples and by 12.31%, 15.92% and 19.68%, respectively, compared with the IF treated samples (Table 1). Regarding the precooling stage, no significant differences were observed between the IF and UIF treated fish samples (p > 0.05). As ultrasonic treatment was not imposed at this stage, the cooling rate was mainly affected by the temperature difference between the freezing medium and the large yellow croaker sample. The phase transition stage is the most important during the freezing process since a particularly large quantity of crystal nuclei are developed and 80% of the liquid water progressively converts into ice crystals [34], [35]. A higher freezing rate can result in the generation of small ice crystals at this phase, which is crucial to obtain high-quality frozen fish and fish products [36], [37], [38]. The freezing time of the phase transition stage was shortened for the TUF treated large yellow croaker samples, as the enhanced ultrasonic cavitation facilitated nucleation and heat transfer during the freezing process.

**Table 1:** The time large yellow croaker muscles spent at each freezing stage as impacted by different freezing treatments.

Treatment	Precooling stage (min)	Phase transition stage (min)	Subcooling stage (min)	Total freezing time (min)
AF	268.17 ± 2.74^a^	167.33 ± 1.21^a^	184.50 ± 1.31^a^	620.17 ± 2.23^b^
IF	39.83 ± 0.71^b^	76.84 ± 0.54^b^	64.83 ± 0.66^c^	181.57 ± 0.95^d^
SUF	37.67 ± 1.09^b^	57.50 ± 1.30^d^	64.03 ± 0.31^c^	159.22 ± 1.16^c^
DUF	35.17 ± 1.44^b^	53.66 ± 1.38^e^	63.84 ± 0.89^b^	152.67 ± 2.42^a^
TUF	35.83 ± 1.27^b^	46.98 ± 1.77^c^	63.02 ± 2.02^b^	145.83 ± 1.71^e^

Results are expressed as mean ± standard deviation (n = 3). The average value with different letters in the same column showed significant differences (p < 0.05). AF, air freezing; IF, immersion freezing; SUF, single-ultrasound assisted freezing; DUF, dual-ultrasound assisted freezing; TUF, triple-ultrasound assisted freezing.

### 3.2 Thawing loss, cooking loss and WHC

**Fig. 4:**
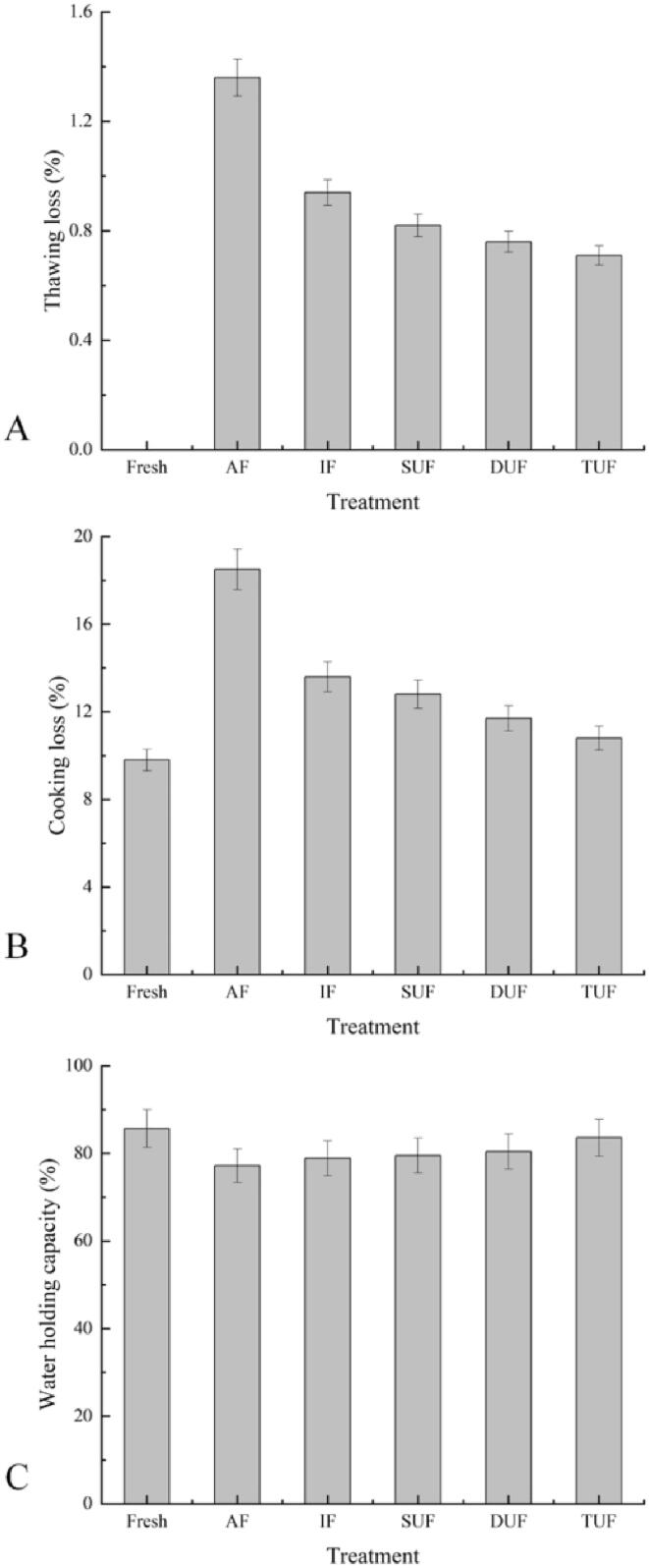
Changes in the thawing loss (A), cooking loss (B) and water holding capacity (C) of large yellow croaker samples under different freezing treatments. Fresh, fresh fish meat; AF, air freezing; IF, immersion freezing; SUF, single-ultrasound assisted freezing; DUF, dual-ultrasound assisted freezing; TUF, triple-ultrasound assisted freezing.

Thawing loss is an important index for evaluating the quality of frozen food and could affect the weight, appearance, colour, and sensory quality of fish and fish products [39], [40]. Thawing loss is related to ice crystal size and distribution, the thawing rate, the degree of water reabsorption, the integrity of muscle tissue, the WHC of the tissue and so on [41]. Fig. 4A shows that the thawing losses of the AF-and IF-treated samples were 1.36% and 0.94%, respectively. After UIF treatment, the thawing loss gradually decreased as the ultrasonic frequency increased. Samples treated with TUF had the lowest thawing loss (0.71%), which was lower than that of the AF and IF treated samples. In addition, the thawing loss of samples treated with TUF was lower than that of the DUF and SUF treated samples. The reduced thawing loss from the UIF treated samples is related to the formation of small and even ice crystals, which results in less damage to the muscle structure. AF-treated samples had the highest thawing loss, and a slow freezing rate could increase the freezing time and contribute to the migration of water to the extracellular space, thus leading to the formation of irregular and large ice crystals and the destruction of muscle structure. Therefore, the AF sample had a weak ability to re-absorb the melted water back into the cells after thawing. The effects of UIF on the freezing speed and quality of common carp (Cyprinus carpio) at different ultrasonic power levels was evaluated by Sun et al. [42]. Their results revealed that UIF treated samples had less thawing loss than AF and IF treated samples, which was similar to the current study.

Cooking loss is primarily attributed to the damage of muscle structure induced by heat-induced denaturation of myofibrillar protein [43]. The change tendency of cooking loss was similar to that of thawing loss. The highest cooking loss (18.49%) was observed in AF treated samples, followed by IF and UIF treated samples (Fig. 4B). The cooking losses of TUF, DUF and SUF treated samples were reduced by 41.62%, 36.76% and 30.81% compared with the AF treated samples, respectively, and reduced by 20.59%, 13.97% and 5.88%, compared with IF treated samples, respectively. The TUF treated large yellow croaker samples showed the lowest cooking loss, as the muscle structure was minimally damaged after being frozen. Structural destruction of muscle protein leads to the loss of free water, thereby reducing cooking loss [44]. WHC is well correlated with the sensory characteristics of fish muscles [45]. The WHCs of the TUF, DUF and SUF treated samples increased by 8.29%, 4.15% and 2.98%, respectively, compared with the AF treated samples (Fig. 4C). TUF treatment effectively decreased the cooking loss and increased the WHC of large yellow croaker, which was closest to the fresh sample with a WHC of 85.70% and a cooking loss of 9.80%. In this study, the effective role of ultrasonic treatment in increasing the WHC might be ascribed to modifying or mechanically destroying the native protein structure, resulting in the exposure of more active groups including reactive sulfhydryl and hydrophobic groups [46].

### 3.3 TPA results

**Fig. 5:**
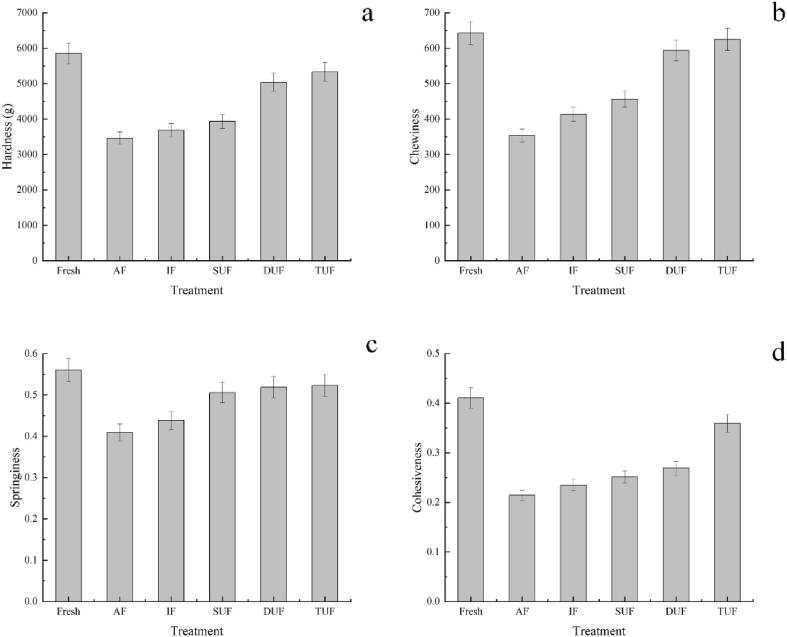
Changes in hardness (a), chewiness (b), springiness (c) and cohesiveness (d) of large yellow croaker samples under different freezing treatments. Fresh, fresh fish meat; AF, air freezing; IF, immersion freezing; SUF, single-ultrasound assisted freezing; DUF, dual-ultrasound assisted freezing; TUF, triple-ultrasound assisted freezing.

Texture attributes, such as hardness, which is associated with the denaturation of muscle structure, are often regarded as freshness indicators for fish [47]. Fig. 5 shows that different ultrasonic frequency treatments had significant effects on the changes in muscle hardness of large yellow croaker samples. The AF treated sample had the lowest hardness (3459.91 g), followed by the IF and UIF treated samples. The TUF treated sample had the highest hardness (5332.98 g), which was closest to the fresh sample with a hardness of 5856.76 g. The chewiness, springiness and cohesiveness of the fish muscle after ultrasonic treatment were greater than those of the AF and IF treatments, and the TUF treated samples had higher chewiness, springiness and cohesiveness than the DUF and SUF treated samples, which can be attributed to the fact that the bubble radius in the TUF field was larger than that of the DUF and SUF fields. Under the same ultrasonic treatment time, TUF produced a more obvious cavitation effect than DUF and SUF. Each ultrasonic frequency in TUF treatment produced a cavitation effect, allowing more air to enter the liquid to form more cavitation nuclei. The increase in WHC could also result in higher values of hardness, chewiness, springiness and cohesiveness [44].

### 3.4 Colour and pH value

Colour is an important indicator affecting the overall appearance and acceptability of fish [48]. The L* values of all frozen large yellow croaker samples were higher than that of the fresh sample (Table 2). The AF-treated samples had a higher thawing loss, resulting in more free water on the fish surface to cause a higher L* value. The L* values of the UIF-treated samples were significantly decreased compared with those of the AF-and IF-treated samples (p < 0.05), and there were no significant differences among the fresh samples and UIF-treated samples (p > 0.05). In particular, the TUF samples had the lowest L* value, which was closest to that of the fresh sample. L* values are influenced by changes in the structure of the muscle due to the water flowing out of the cells during the freezing process. The cavitation bubbles produced in the freezing medium by ultrasonic treatment burst to form micro-jets, which increased the flow velocity of the freezing medium and promoted heat transfer between the freezing medium and the fish, thereby improving the freezing efficiency and effectively protecting the muscle structure. Farouk et al. [49] reported that slowly frozen meat was lighter in colour than rapidly frozen meat. The more free water on the fish surface was brought on by a higher thawing loss, resulting in greater light reflection and a lighter colour [50], which was in accordance with the results of the thawing loss analysed above. Monteschio et al. [51] showed that structural changes, such as disruption of protein conformation, could increase the light dispersion, leading to an increase in the L* value. However, there were no significant differences in the a* and b* values among all the samples (p > 0.05). Lind et al. [52] also stated that freezing speed had no significant effect on the a* value in lambs.

**Table 2:** The color and pH values of large yellow croaker muscles with different freezing treatments.

Treatment	Color			pH
	L*	a*	b*	
Fresh	47.74 ± 0.76^c^	5.44 ± 0.25^a^	4.55 ± 0.71^a^	6.99 ± 0.03^a^
AF	53.13 ± 0.14^a^	5.65 ± 0.36^a^	4.13 ± 1.33^a^	6.85 ± 0.04^b^
IF	50.74 ± 0.91^a^	5.43 ± 0.61^a^	4.44 ± 0.16^a^	6.86 ± 0.01^b^
SUF	48.79 ± 2.09^bc^	5.30 ± 0.10^a^	4.49 ± 0.81^a^	6.87 ± 0.01^b^
DUF	48.57 ± 3.44^c^	5.48 ± 0.38^a^	4.09 ± 0.69^a^	6.87 ± 0.02^b^
TUF	47.96 ± 1.97^bc^	5.43 ± 0.77^a^	4.59 ± 0.92^a^	6.88 ± 0.01^b^

The means in the same column with different letters differ significantly (p < 0.05). Fresh, fresh fish; AF, air freezing; IF, immersion freezing; SUF, single-ultrasound assisted freezing; DUF, dual-ultrasound assisted freezing; TUF, triple-ultrasound assisted freezing.

The polar groups in the proteins are attracted to each other as the pH value reaches the isoelectric point of the dominant in fish proteins, which can lead to a reduction in WHC and affect the quality of fish [53]. As indicated in Table 2, the pH values of all the thawed large yellow croaker samples were lower than that of the fresh sample, which can be related to the accumulation of inorganic phosphoric acid and lactic acid and ATP degradation in the process of freezing and thawing [54]. However, no significant differences were observed in the pH values among all the thawed samples (p > 0.05). The freezing time in this study was too short to lead to a significant change in pH value. Thus, the pH value may not be a key factor resulting in changes in thawing and cooking losses. Zhang et al. [11] evaluated the effects of UIF on the quality of porcine longissimus muscles and reported similar results, where they found that the pH of the samples did not differ significantly (p > 0.05) under different UIF conditions.

### 3.5 Analysis by LF-NMR

**Fig. 6:**
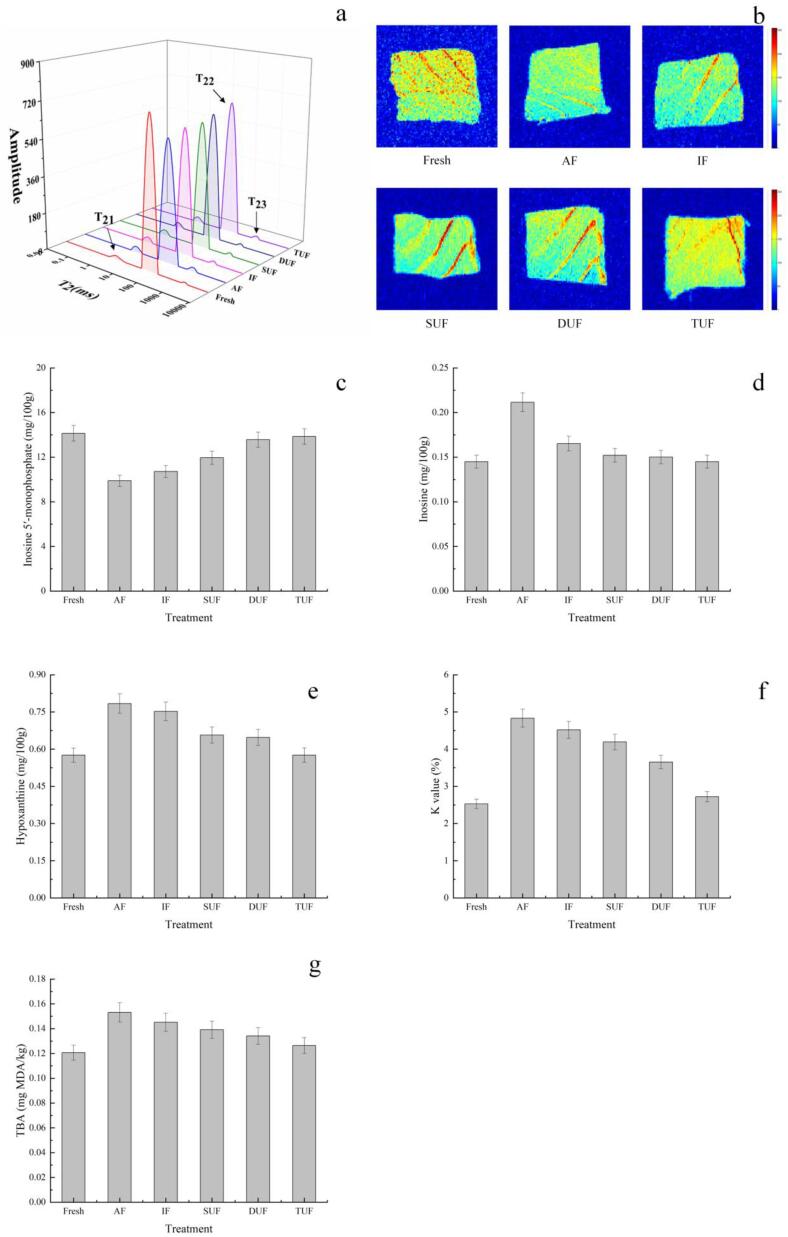
Changes in water distribution (a), magnetic resonance imaging (b), inosine 5′-monophosphate (IMP) (c), inosine (HxR) (d), hypoxanthine (Hx) (e), K values (f), and thiobarbituric acid reactive substances (TBARS) (g) of large yellow croaker samples under different freezing treatments. Fresh, fresh fish meat; AF, air freezing; IF, immersion freezing; SUF, single-ultrasound assisted freezing; DUF, dual-ultrasound assisted freezing; TUF, triple-ultrasound assisted freezing. (For interpretation of the references to color in this figure legend, the reader is referred to the web version of this article.)

LF-NMR and magnetic resonance imaging (MRI) could assess the internal distribution of water and mobility of frozen muscle to understand water migration as a result of different freezing treatments [55], [56]. There are three peaks corresponding to the three relaxation components, known as T_21_ (<10 ms), T_22_ (20–400 ms) and T_23_ (>1000 ms), which represent bound water, immobilized water and free water, respectively. pT_21_, pT_22_ and pT_23_ correspond to the areas of T_21_, T_22_ and T_23_, respectively. There was no significant difference in pT_21_ between the TUF treated sample and fresh sample (Fig. 6a), which indicated that UIF treatment had no obvious destructive effects on the impaired capillaries in large yellow croaker [57]. The damaged myofibrils could hardly absorb the melted water outside the cell due to cell membrane destruction, resulting in the partially immobilized water being converted to free water. Cai et al. [58] showed that red seabream fillet thawed by microwave irradiation had more myofibril damage, leading to the loss of more immobilized water from the muscle compared with the fresh sample. In the UIF treated samples, the immobilized water content increased as the ultrasonic frequency increased, and the TUF-treated sample had the highest immobilized water content because TUF treatment maintained or even increased intracellular water to improve the quality of large yellow croaker. However, the AF treated sample had the highest free water content, resulting in a decrease in the elasticity and an increase in the thawing loss of the fish [59], which were consistent with the TPA and thawing loss results.

MRI can visually observe the spatial and internal morphology and molecular distribution of water in large yellow croaker. Red is the region with high proton density, and blue is the region with low proton density [60]. The colour of the AF treated sample was bluer than that of the other samples (Fig. 6b), suggesting that the AF treated sample had more intensive degradation and destruction of the microstructure of the myofibrils. The UIF treated samples had more red areas than the AF and IF treated samples, especially the TUF treated sample, which also suggested that TUF had optimal water conservation and maintained the quality of the large yellow croaker samples during the freezing process. However, there were no significant differences among UIF treated samples. It is worth noting that the MRI results were consistent with the changes in LF-NMR relaxation time.

### 3.6 ATP-related compounds and K value

The sequence of ATP degradation in fish muscle is ATP → ADP → AMP → IMP → HxR → Hx [61]. IMP provides a fleshy and sweet flavour and contributes to an improvement in fish quality, whereas its transformation to HxR and Hx results in unpleasant bitterness and off flavours that contribute negatively to the flavour of fish [62]. The initial IMP concentration was 14.14 mg/100 g (Fig. 6c) and decreased in all frozen samples with the action of autolytic enzymes [63]. The UIF treated samples increased the freezing speed to protect the structure and quality of muscles to reduce the degradation of IMP. The TUF treated sample had the highest IMP concentration, showing an increase of 40.18% and 29.26% compared to the AF and IF treated samples, respectively. However, there were no significant differences (p > 0.05) among UIF treated samples probably due to the short freezing time. Li et al. [64] also reported that the initial HxR and Hx concentrations were 0.1450 and 0.5759 mg/100 g, respectively, and both of these concentrations increased as the IMP concentration decreased in common carp (Cyprinus carpio) during the freezing process

The K-values of AF treated samples (4.83%) were significantly higher than those of fresh samples (2.53%). The UIF treatments could delay the increase in the K value of large yellow croaker samples. The K values of the SUF, DUF and TUF treated samples decreased by 13.23%, 24.39% and 43.67%, respectively, compared with the AF treated samples and 7.21%, 19.14% and 39.76%, respectively, compared with the IF treated samples. The K-values gradually decreased with increasing ultrasonication frequency, and the K-values of the TUF-treated samples (2.72%) were lower than those of the SUF (4.19%) and DUF (3.65%) treated samples. Phosphorylase and cathepsin are the key factors that cause ATP degradation. UIF could induce cavitation effects during propagation and the high temperature and high pressure generated by the bursting of the cavitation bubbles could both result in a reduction in enzymatic activity [65].

### 3.7 Analysis of TBARS

Although the biochemical reactions are largely inhibited by the freezing process, several non-negligible oxidative degradations remain. The TBARS values of fresh and AF-treated samples were 0.12 and 0.15 mg MDA/kg, respectively (Fig. 6g). The rate of fat oxidation can accelerate during the freezing process, as ice crystals could cause damage to cells and lead to the release of oxidants, particularly non-haem iron [66], [67]. The formation and development of large ice crystals destroyed the integrity of muscle cells, and intracellular oxidative precursors were released to accelerate fat oxidation [68]. However, the TBARS values of the UIF treated samples were lower than those of the AF and IF treated samples. The TBARS values of SUF, DUF and TUF treated samples decreased by 9.17%, 12.39% and 17.42% compared with the AF treated samples, respectively, and by 4.15%, 7.55% and 12.86% compared with the IF treated samples, respectively. It should be noted that the TBARS value of the TUF treated sample (0.13 mg MDA/kg) was close to that of the fresh sample. TUF treatment produced fine and uniformly distributed ice crystals in muscle tissue through relatively rapid freezing, which better protected the cell structure and muscles quality. In addition, UIF could effectively inhibit lipid oxidation by reducing the activities of lipase, phospholipase and lipoxygenase [69]. Therefore, UIF treatment decreased the TBARS value of the frozen large yellow croaker compared with that of other freezing treatments. Zhang et al. [48] found that porcine longissimus muscles after UAF at 180 W had significantly lower lipid oxidation than that of the AF and IF treated samples during frozen storage (p > 0.05). However, Shi et al. [19] pointed out that UAF significantly increased the TBARS value in fish (p < 0.05), which may be because most of the water converts to ice at low temperature during the IF process, weakening the cavitation effect.

### 3.8 Light microscopy observations

**Fig. 7:**
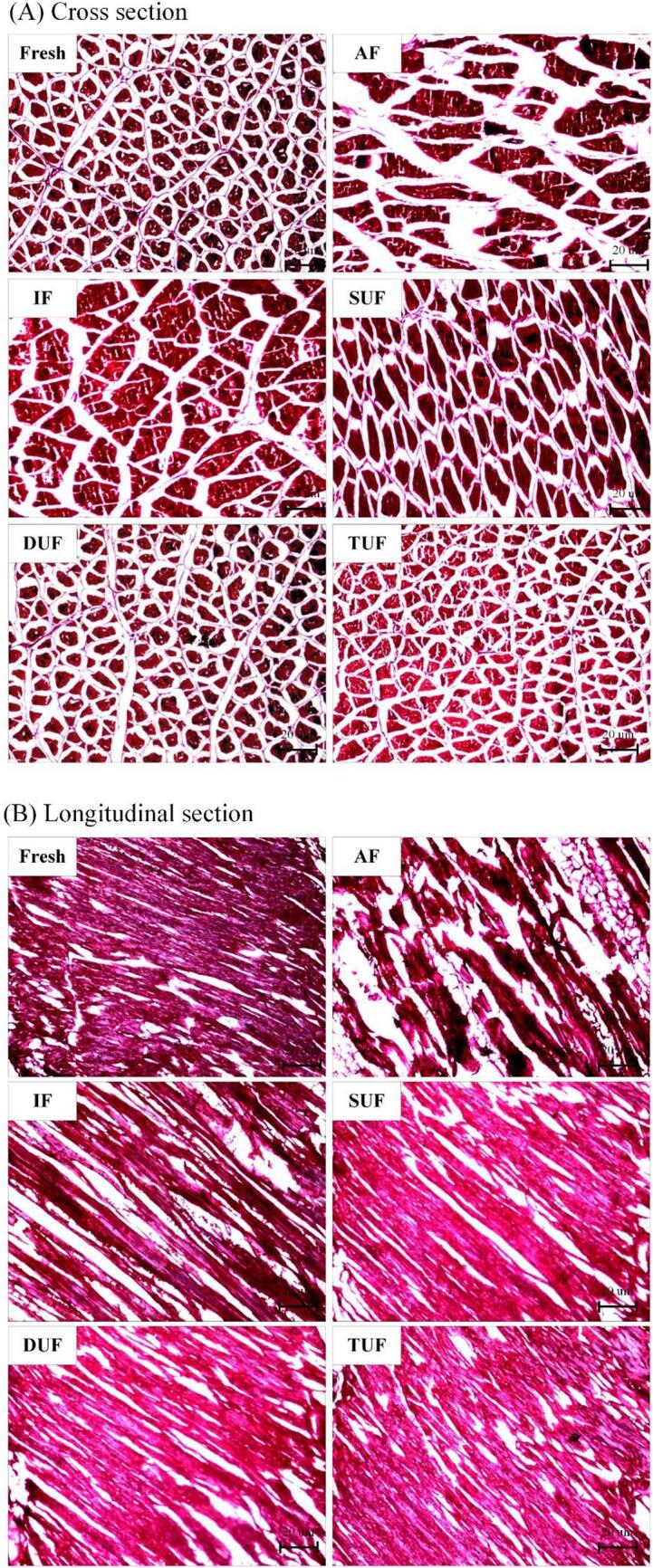
Changes in the microstructure cross section (A) and longitudinal section (B) of large yellow croaker muscles samples under different freezing treatments. Fresh, fresh fish meat; AF, air freezing; IF, immersion freezing; SUF, single-ultrasound assisted freezing; DUF, dual-ultrasound assisted freezing; TUF, triple-ultrasound assisted freezing. (For interpretation of the references to color in this figure legend, the reader is referred to the web version of this article.)

The muscle myofibrils of the fresh sample were neatly arranged and dense (Fig. 7A and 7B); however, the muscle myofibrils of AF and IF treated samples were irregularly arranged, presented seriously twisted myofibrils, had a great majority of broken muscle myofibrils and the gaps were enlarged due to the slower freezing rate. The TUF treated samples appeared similar to the fresh sample. The muscle myofibrils of the TUF treated samples were neatly aligned and appeared to be tighter and denser. The LF-NMR results also showed that the AF and IF treated samples had more free water and that the muscle structure was destroyed, leading to free water entering the gaps of the myofibrils. The TUF treated samples had more immobilized water and a more complete microstructure, which could be due to the cavitation effect generated by UIF treatment [14]. The cavitation bubbles reaching the critical nucleus size could act as nuclei and induce the formation of ice crystals. The collapse of cavitation bubbles causes the pre-existing ice crystals to break into smaller sizes, which promotes secondary nucleation and produces more ice cores and small ice crystals [70]. In addition, the microstreamin g generated by the movement of cavitation bubbles can enhance heat and mass transfer. All of these factors might contribute to the accelerated freezing rate and facilitate the formation of small and uniform ice crystals, thus effectively improving the microstructure of large yellow croaker during the freezing process. Zhang et al. [71] demonstrated that ultrasound generated microflows to promote nucleation during freezing. A particular intensity of ultrasound could result in the breakdown of ice crystals, thus resulting in a reduction in the ice crystal size [72]. Small ice crystals and their uniform distribution during the freezing process were found to be beneficial for the quality characteristics of frozen foods [73].

## 4 Conclusions

This research evaluated the effects of multi-frequency ultrasonic treatments on the freezing rate, quality properties and structural characteristics of large yellow croaker. The results showed that multi-frequency UIF significantly accelerated the freezing rate of the frozen samples, and TUF-treated samples had the fastest freezing rate. These effects become more obvious with an increase in the number of ultrasonic frequencies. Therefore, TUF treated samples retained better quality attributes than the DUF and SUF treated samples, as multi-frequency ultrasonic treatment generated more cavitation nuclei and enhanced the cavitation effects. The samples treated with TUF had a higher water holding capacity and immobilized water content, lower thawing and cooking losses, and better texture characteristics and microstructure, which were close to those of the fresh samples. The light microscopy observation images demonstrated that TUF treatment facilitated the formation of smaller and more uniform ice crystals. However, the freezing conditions had no significant influence on the colour or pH values. These results indicated that multi-frequency ultrasonic treatment is a promising way to effectively accelerate the freezing rate and improve the physicochemical properties and structural characteristics of large yellow croaker.

## CRediT authorship contribution statement

**Xuan Ma:** Conceptualization, Data curation, Formal analysis, Investigation, Methodology, Software, Writing - original draft. **Jun Mei:** Conceptualization, Data curation, Investigation, Methodology, Project administration, Validation, Writing - original draft, Writing - review & editing. **Jing Xie:** Funding acquisition, Methodology, Project administration, Validation, Writing - review & editing.

## Declaration of Competing Interest

The authors declare that they have no known competing financial interests or personal relationships that could have appeared to influence the work reported in this paper.
